# The association of serum vitamin D with incident diabetes in an African American population

**DOI:** 10.1038/s41387-022-00220-4

**Published:** 2022-10-13

**Authors:** Joshua J. Joseph, Susan Langan, Joseph Lunyera, Bjorn Kluwe, Amaris Williams, Haiying Chen, Michael C. Sachs, Kristin G. Hairston, Alain G. Bertoni, Willa A. Hsueh, Sherita H. Golden

**Affiliations:** 1 Division of Endocrinology, Diabetes and Metabolism, The Ohio State University Wexner Medical Center, Columbus, OH USA; 2 Division of Endocrinology, Diabetes and Metabolism, Johns Hopkins University School of Medicine, Baltimore, MD USA; 3 Unit of Biostatistics, Duke University School of Medicine, Durham, NC USA; 4 Division of Public Health Sciences, Wake Forest School of Medicine, Winston-Salem, NC USA; 5 Department of Medical Epidemiology and Biostatistics, Karolinska Institutet, Stockholm, Sweden

**Keywords:** Type 2 diabetes, Sterols, Nutrition, Disease prevention

## Abstract

**Background:**

Incident diabetes risk is inversely proportional to 25-hydroxyvitamin D [25(OH)D] levels among non-Hispanic white but is unclear among African American (AA) populations. Serum 25(OH)D2 may be an important component of total 25(OH)D among AA populations due to higher levels of melanin.

**Objective:**

To assess the association of serum 25(OH)D with incident diabetes among AAs and stratify by detectable 25(OH)D2.

**Design:**

Serum 25(OH)D2 and 25(OH)D3 were collected from 2000 to 2004 among AA participants in the Jackson Heart Study. A cosinor model was used to adjust for the seasonality of 25(OH)D3; 25(OH)D3 and 25(OH)D2 were combined to ascertain total 25(OH)D. Incident diabetes (fasting glucose ≥126 mg/dl, use of diabetes drugs, or HbA1c ≥6.5%) was assessed over 12 years among adults without diabetes at baseline. Participants with missing baseline covariates or diabetes follow-up were excluded. Hazard ratios (HR) were estimated using Cox modeling, adjusting for age, sex, education, occupation, smoking, physical activity, alcohol use, aldosterone, and body-mass index.

**Results:**

Among 3311 adults (mean age 53.3 years, 63% female) 584 participants developed diabetes over a median of 7.7 years. After adjustment, 25(OH)D ≥20 compared to <12 ng/ml was associated with a HR 0.78 (95% CI: 0.61, 1.00). Among participants with detectable 25(OH)D2 and 25(OH)D3 (*n* = 1671), 25(OH)D ≥ 20 ng/ml compared to <12 ng/ml was associated with a 35% (HR 0.65, 95% CI: 0.46, 0.91) lower risk of diabetes.

**Conclusions:**

Higher levels of 25(OH)D may be protective against the development of diabetes among AA individuals, particularly among those with detectable 25(OH)D2 and 25(OH)D3.

## Introduction

Vitamin D is a fat-soluble paracrine and endocrine signaler important for bone, glycemic and general health [1]. Vitamin D3 is produced from ultraviolet light acting on 7-dehydrocholesterol in the skin to produce previtamin D3 [1], which is converted to the less active precursor 25-hydroxyvitamin D (25[OH]D), and then enzymatically converted to the active form of the vitamin, 1,25- dihydroxyvitamin D (1,25[OH]D) by 1-alpha hydroxylase in the kidney and other tissues [1]. Smaller amounts may be obtained from the diet (25[OH]D2) or supplements (25[OH]D2 or 3), which may be of greater importance in persons with little sunlight exposure. Vitamin D2 originates from certain fungi and can only be obtained from food or supplements. Although 25(OH)D2 represents a smaller fraction of total 25(OH)D, it binds the human vitamin-D binding protein with less avidity than 25(OH)D3, and thus may be more biologically active [2]. 25(OH)D2 may be more important among populations with darker skin pigmentation, as melanin absorbs the ultraviolet radiation and leads to less vitamin D3 synthesis, for a given exposure compared to less pigmented skin [3]. Similar to vitamin D3, Vitamin D2 is metabolized into 25(OH)D in the liver. Although 1,25(OH)D is the biologically active form, the more stable 25(OH)D is measured in serum to assess vitamin-D sufficiency. The role of vitamin D in calcium and bone metabolism is well established, but there is increasing interest in the anti-inflammatory and metabolic effects of vitamin D given the rising prominence of type 2 diabetes mellitus (diabetes) and obesity [1, 4, 5].

Pre-clinical studies reveal a link between vitamin D and insulin secretion [6]. Pancreatic ß-cells express vitamin-D receptors. Vitamin D may influence insulin activity and insulin sensitivity through a number of mechanisms: (1) intracellular calcium levels determine the ability of insulin-sensitive cells to conduct insulin-dependent activities, and vitamin D may regulate calcium flux between the extra- and intracellular milieu of pancreatic ß-cells; (2) vitamin D may stimulate the expression of insulin receptors in insulin-sensitive tissue; (3) vitamin D may activate PPAR-δ, which in turn regulates fatty acid metabolism in muscle and adipose tissue [7].

Evidence for an inverse association of vitamin D with incident diabetes comes from observational cohort studies, interventional trials, and meta-analyzes with the majority non-Hispanic white (NHW) participants [8–12]. There are limited data on the role of vitamin D in the development of diabetes in African American (AA) populations. In the Atherosclerosis Risk in Communities (ARIC) study, Reis et al. found a negative association of vitamin D with incident diabetes among NHWs, but no association among AA participants [13]. These findings were consistent with previous studies of vitamin D and cardiovascular disease, which revealed a negative association of 25(OH)D with vascular calcification and coronary heart disease (CHD) in European Americans but no association in the AA population [14]. A potential explanation for these findings may be related to genetic differences in the prevalence of vitamin D binding protein (DBP) genotypes by race/ethnicity which impact levels of DBP and therefore bioavailability of vitamin D [15, 16].

Given the conflicting data on the role of 25(OH)D in the development of diabetes in the AA population compared to NHWs, we examined the association of 25(OH)D with incident diabetes in the Jackson Heart Study (JHS). We hypothesized: (1) serum levels of 25(OH)D at baseline will be negatively associated with the risk of incident diabetes during follow-up, (2) the association of categorically higher levels of baseline 25(OH)D with incident diabetes based on Institute of Medicine (IOM) and Endocrine Society (ES) guidelines would yield similar findings, and (3) detectable 25(OH)D2 may influence the association of 25(OH)D with incident diabetes.

## Methods

### Study participants

The JHS is a prospective study of the development and progression of cardiovascular disease in a cohort of 5301 AA adults, aged 21–94 years from the tri-county area of metropolitan Jackson, Mississippi. Enrollment and baseline examinations were performed between 2000 and 2004 with two subsequent in-person follow-up examinations in 2005–2008 and 2009–2013. Details about the study design, recruitment and methods are described elsewhere [17]. Participants were excluded if they had diabetes at baseline (*n* = 1152), or were missing data on baseline or follow-up diabetes status (*n* = 769) or total 25(OH)D (*n* = 69). After these exclusions, 3311 participants were included in the analysis (Supplemental Fig. 1) [18]. The JHS was approved by the institutional review boards of the participating institutions and written informed consent was obtained from all subjects.

### Exposure: 25(OH)D2, 25(OH)D3, total 25(OH)D, and vitamin-D binding protein genotypes

Fasting blood samples were drawn in the supine position and processed using a standardized protocol. Briefly, plasma and serum were prepared from samples by sedimentation in a refrigerated centrifuge within 2 h of blood collection, stored at −70 °C, and sent to central laboratories (University of Minnesota) [17, 19]. Serum 25(OH)D2 and 25(OH)D3 levels were analyzed using Liquid Chromatography-tandem Mass Spectrometry (LC-MS/MS) from stored frozen serum. Detailed methods are included in the Supplemental Methods [20]. The inter-assay coefficient of variation for 25(OH)D2 was 4.9–6.3% at concentrations of 11.4–32.6 ng/mL and 4.6–6.7% at concentrations of 11.3–30.3 ng/mL for 25(OH)D3. The intra-assay coefficient of variation for 25(OH)D3 was 7.16%.

A seasonal pattern was evident for 25(OH)D3, but not for 25(OH)D2 levels, thus we used a cosinor model to adjust for the seasonality of 25(OH)D3 over the calendar year. In this model, 25(OH)D concentration is the dependent variable and the sine and cosine of calendar date (t, 1–365) are the independent variable: 25(OH)D = βo + β_1_*cos(2*π*t/365) + β_2_*sin(2*π*t/365) + (residual) [21]. The period was set to 365 days so that the seasonal curve had one peak (in the summer) and one trough (in the winter) during the year. The cosinor model tested for any effect of age, sex, BMI, and physical activity on the mean and amplitude of the annual sinusoidal pattern of 25(OH)D3. The mean annualized predicted values of 25(OH)D3 were significantly higher for men and for persons of normal BMI compared to overweight or obese persons, and thus mean annualized estimates were adjusted for sex and BMI categories. The measured value of 25(OH)D2 was added to the cosinor model annualized estimate of 25(OH)D3 to obtain an annualized estimate of total 25(OH)D. 25(OH)D ranged from a minimum of 0.10 ng/ml to a maximum of 54.05 ng/ml. Baseline serum 25(OH)D was examined continuously using the standard deviation of log-transformed 25(OH)D. We used 2 categorizations for 25(OH)D: (1) <12 (deficient), 12–19.9 (insufficient), and ≥20 ng/ml (sufficient) based on 2011 IOM guidelines [22] and (1) <20 ng/ml (deficient), 20–29.9 (insufficient) and ≥30 ng/ml (sufficient) based on ES Clinical Practice Guideline on Evaluation, Treatment, and Prevention of Vitamin-D Deficiency [23]. Genotype data for JHS participants, including two single-nucleotide polymorphisms (SNP) in the DBP genotype, rs4588 and rs7041, are derived from Affymetrix 6.0 genotyping platform. Details regarding the genotype-calling algorithm and quality controls have been described elsewhere [24].

### Outcome: diabetes status, estimated β-cell function, and insulin resistance

Fasting glucose and insulin concentrations were measured on a Vitros 950 or 250, Ortho-Clinical Diagnostics analyzer (Raritan, NJ) using standard procedures that met the College of American Pathologists accreditation requirement [19]. A high-performance liquid chromatography system (Tosoh Corporation, Tokyo, Japan) was used to measure glycosylated hemoglobin A1c (HbA1c) concentrations. Diabetes was defined as HbA1c ≥6.5% (48 mmol/mol), fasting blood glucose ≥126 mg/dl, or taking diabetes medications [25]. Though the 2021 American Diabetes Association’s guidelines recommend the use of metformin in those with prediabetes [26], a national sample between 2005 and 2012 found that the use of metformin in those with prediabetes was only 0.7% [27]. Therefore, the risk of misclassification of individuals with prediabetes using metformin was low. Persons without diabetes at baseline who met the criteria for diabetes at one of the two subsequent exams were considered to have incident diabetes.

### Baseline assessments

Baseline information was obtained during clinic visits or at home using standardized questionnaires including demographics, occupation (management/professional versus not), level of education (≥ Bachelor’s degree versus < Bachelor’s degree), tobacco use (current smoking versus not), alcohol use (in the past 12 months versus not) medical conditions and current prescription medication usage. Calibrated devices were used by certified technicians and nurses to measure participants’ weight, waist circumference (average of two measurements around the umbilicus) and height. BMI was calculated as weight (kilograms)/height^2^ (meters). Hypertension was defined as systolic blood pressure ≥140 mmHg, diastolic blood pressure ≥90 mmHg or the use of antihypertensive therapy. Serum aldosterone was measured by radioimmunoassay (Siemens) and the intra-assay coefficients of variation are reported elsewhere [28].

Physical activity in minutes (min) per week was defined according to the American Heart Association (AHA) categorization [29] as poor health (0 minutes of moderate and vigorous activity), intermediate health (>0 min but <150 min of moderate activity), (>0 min but <75 min of vigorous activity or >0 min but <150 min of combined moderate and vigorous activity) and ideal health (>150 min of moderate activity, >75 min of vigorous activity or >150 min of combined moderate and vigorous activity). Dietary intake was defined according to the AHA categorization [29] using a validated 158-item food frequency questionnaire administered face to face by trained AA interviewers [30]. The questionnaire had some slight differences compared to the AHA categorization regarding units of servings, which required modification of the metrics. Components of the ideal diet were: fruits and vegetables ≥4.5 cups/day, fish ≥two 3.5 ounce servings per week (non-fried), fiber-rich whole grains ≥three 1 ounce-equivalent servings/day, sodium <1500 mg/day and sugar-sweetened beverages ≤450 kcal (36 ounces)/week. Participants were given 1 point per dietary component at goal for a total score ranging from 0 to 5. Participants were classified as ideal (4 or 5 of the 5 metrics), intermediate (2 or 3 out of the 5 metrics) or poor (0 or 1 out of the 5 metrics). Insulin resistance and β-cell function were estimated using homeostasis model assessment of insulin resistance (HOMA-IR) = (fasting plasma glucose [mmol/L] × fasting plasma insulin [mU/mL]) ÷ 22.5 and β-cell function (HOMA-β) = (20 × fasting plasma insulin) ÷ (fasting plasma glucose – 3.5)% [31].

### Statistical analysis

Total 25(OH)D values were log-transformed prior to continuous analyzes due to a non-normal distribution. To explore potential non-linear relationships and evaluate for dose-response relationships, a non-log-transformed total 25(OH)D was categorized as described above. Descriptive statistics were used to compare the baseline characteristics of participants by categories of total 25(OH)D using one-way analysis of variance for normally distributed continuous variables, Mann–Whitney and Kruskal–Wallis tests for non-normally distributed continuous variables, and the Chi-square test for categorical variables.

We defined the time of incident of diabetes as the midpoint between last exam without diabetes and the exam at which diabetes was detected [28]. For participants who remained free of diabetes, the follow-up time was censored at their last available visit. Cox proportional hazards modeling was utilized to estimate hazard ratios (HR) for incident diabetes after confirming proportionality using Schoenfeld residuals. Sequential multivariable adjustment modeling was performed: Model 1: age, sex, education, current occupation status; Model 2: Model 1 + smoking, AHA physical activity, alcohol use, and aldosterone; Model 3: Model 2 + BMI (kg/m^2^). We performed sensitivity analyses, including analyzing 25(OH)D2 and 25(OH)D3 individually and limiting the analytic sample to participants with detectable 25(OH)D2 to confirm the robustness of our results. To test for possible effect modification of the relationship between 25(OH)D and incident diabetes by DBP genotype, we added interaction terms to the models. The likelihood ratio test was used to determine evidence of a statistically significant interaction. Statistical significance was defined as two-sided alpha <0.05 in the main analysis and <0.10 for interactions [32]. Analyzes were performed using Stata 13.1 (Statacorp, College Station, TX).

### Preprint

A previous version of this manuscript was published as a preprint.

## Results

### Baseline characteristics

**Fig. 1:**
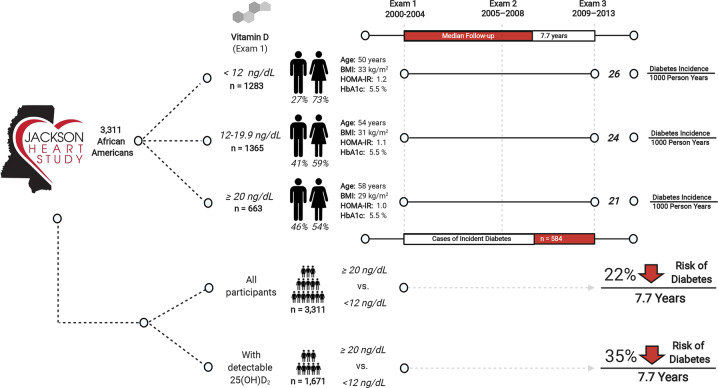
The association of serum vitamin D with incident diabetes in an African American population. The figure presents the baseline characteristics by 25(OH) vitamin-D categories and shows the association of 25(OH)D with incident diabetes by 25(OH)D2 and 25(OH)D3 status.

The baseline characteristics of the 3311 participants across 25(OH)D categories are presented in Table 1 and Supplemental Table 1 [18]. Participants in higher categories of 25(OH)D were older with lower BMI, waist circumference, HOMA-IR, and higher levels of aldosterone, physical activity, and healthier dietary intake (all comparisons, *P* < 0.01; Fig. 1). Thirty-nine percent and 80% of the cohort had a 25(OH)D in the deficient range by IOM guidelines (<12 ng/ml) and ES guidelines (<20 ng/ml), respectively. Less than 39% and 3% of the cohort had a 25(OH)D in the sufficient range by IOM guidelines (≥20 ng/ml) and ES guidelines (≥30 ng/ml), respectively. All participants had detectable 25(OH)D3. Almost half of the participants had an undetectable 25(OH)D2 level (Supplemental Table 2) [18].

**Table 1:** Baseline characteristics of Jackson Heart Study Participants by 25(OH) vitamin-D categories (Institute of Medicine).

		Vitamin-D concentration (ng⁄mL)			
Baseline characteristics^a^	All, *n* = 3311	Less than 12, *n* = 1283	12–19.9, *n* = 1365	20+, *n* = 663	*P* value
Age	53.31 (12.48)	50.26 (12.02)	53.98 (12.34)	57.80 (12.09)	<0.0001
Female, sex (%)	63.30	72.72	58.75	54.45	<0.0001
Education >bachelor's degree (%)	37.03	36.94	35.24	40.87	0.0477
Occupation, management/professional (%)	38.95	39.36	37.36	41.45	0.1942
Current smoking (%)	11.96	13.27	12.41	8.51	0.0076
Current alcohol use (%)	49.98	51.21	49.15	49.32	0.5317
AHA physical activity^b^					<0.0001
Poor (%)	45.05	49.18	44.47	38.22	
Intermediate (%)	33.53	32.42	34.87	32.93	
Ideal (%)	21.42	18.39	20.66	28.85	
AHA dietary intake^b^					<0.0001
Poor (%)	63.79	68.43	64.25	53.85	
Intermediate (%)	35.52	31.18	35.16	44.65	
Ideal (%)	0.69	0.39	0.59	1.51	
Vegetables (servings/day)	1.46 (0.69)	1.48 (0.7)	1.45 (0.7)	1.45 (0.68)	0.2385
Fruit (servings/day)	1.53 (1.17)	1.50 (1.32)	1.51 (1.04)	1.61 (1.11)	0.0004
Body-mass index (kg/m^2^)^c^	31.20 (7.04)	32.62 (7.79)	30.76 (6.62)	29.34 (5.65)	<0.0001
Waist circumference (cm)^d^	98.65 (15.64)	100.60 (17.1)	98.16 (14.97)	95.86 (13.36)	<0.0001
Systolic blood pressure (mmHg)	124.97 (17.43)	123.78 (16.87)	125.36 (17.72)	126.47 (17.77)	0.0020
Diastolic blood pressure (mmHg)	79.37 (10.37)	79.22 (10.36)	79.74 (10.49)	78.88 (10.14)	0.1014
Glucose (mg/dl)	90.30 (8.9)	90.50 (8.97)	90.11 (8.94)	90.30 (8.7)	0.5624
Hemoglobin A1c %^e^	5.50 (0.47)	5.50 (0.47)	5.49 (0.48)	5.52 (0.43)	0.7440
ln HOMA-IR^f^	1.11 (0.55)	1.17 (0.55)	1.10 (0.55)	1.02 (0.55)	<0.0001
ln HOMA-β^f^	5.28 (0.51)	5.33 (0.51)	5.28 (0.52)	5.18 (0.49)	<0.0001
Aldosterone (ng/dl)	5.49 (4.6)	5.16 (5)	5.59 (4.4)	5.91 (4.12)	<0.0001
hs-CRP (mg/dl)^g^	0.47 (0.71)	0.55 (0.77)	0.41 (0.64)	0.43 (0.72)	<0.0001
Diabetes incidence rate/1000 PY	24.33	26.19	24.39	20.66	

^a^Mean (SD) or percentages are listed, *P* values were calculated using two (categorical variables), ANOVA (parametric continuous variables), and Kruskal–Wallis test (nonparametric continuous variables).

^b^Physical activity and dietary intake recommendations were defined by AHA 2020 guidelines. Physical activity was considered ideal if the participant achieved 150 min/wk or greater of moderate-intensity or 75 min/wk or greater of vigorous-intensity physical activity [29]. Dietary intake was considered ideal if the participant met four to five of the five following recommendations: fruits and vegetables of 4.5 cups/d or more; fish of two 3.5-oz servings per week or more (preferably oily fish); fiber-rich whole grains of three 1-oz-equivalent servings per day or more; sodium 1500 mg/d or less; and sugar-sweetened beverages of 450 kcal (36 oz)/wk or less [29].

^c^Body-mass index—*n* = 3311 (<12 *n* = 1283, 12–19.9 *n* = 1365, 20+ *n* = 663).

^d^Waist circumference—*n* = 3311 (<12 *n* = 1283, 12–19.9 *n* = 1365, 20+ *n* = 663).

^e^Hemoglobin A1c—*n* = 3261 (<12 *n* = 1261, 12–19.9 *n* = 1348, 20+ *n* = 652).

^f^HOMA-IR and HOMA-β—*n* = 3188 (<12 *n* = 1248, 12–19.9 *n* = 1303, 20+ *n* = 637).

^g^High sensitivity-C reactive protein—*n* = 3310 (<12 *n* = 1282, 12–19.9 *n* = 1365, 20+ *n* = 663).

### Longitudinal assessments

During a median follow-up of 7.7 years, 584 participants developed diabetes (incidence rate 23.1 per 1000-person years). There was a graded decrease in incidence rates across higher IOM 25(OH)D categories (<12, 12–19.9, and ≥20 ng/ml): 26.2, 24.4, and 20.7 per 1000-person years from lowest to highest category (*P* < 0.0001) (Table 1), with a similar graded association across ES Guidelines (Supplemental Table 1) [18].

### Association of total 25(OH)D with incident diabetes among all participants (*n* = 3311)

The unadjusted and adjusted HRs for the association of 25(OH)D with incident diabetes categorically and continuously are presented in Table 2 and Fig. 1. After adjustment for covariates including age, sex, education, occupation, smoking, physical activity, alcohol use and aldosterone, 25(OH)D ≥20 compared to <12 ng/ml (IOM) was associated with a 31% (HR 0.69, 95% CI: [0.54, 0.89]) lower risk of diabetes. Adjustment for BMI attenuated these findings (HR 0.78, 95% CI: 0.61, 1.00). Using ES guidelines, 25(OH)D ≥30 compared to <20 ng/ml was associated with a 51% (HR 0.49, 95% CI: 0.24, 0.99) lower risk of diabetes, with BMI attenuating these findings. Every 1-log standard deviation (SD) increase in 25(OH)D was associated with a 10% (HR 0.90, 95% CI: 0.83–0.98) lower risk of diabetes which became nonsignificant after adjustment for BMI.

**Table 2:** The association of total 25(OH) vitamin D with incident diabetes.

Institute of Medicine Guidelines	Cox proportional hazards model—hazard ratio (95% CI) of all participants (*n* = 3311)			
25(OH) vitamin-D categories	Unadjusted	Model 1	Model 2	Model 3
<12	Referent	Referent	Referent	Referent
12–19.9	0.92 (0.77–1.11)	0.85 (0.71–1.02)	0.86 (0.71–1.03)	0.92 (0.77–1.12)
20+	0.78 (0.61–0.98)	0.67 (0.53–0.86)	0.69 (0.54–0.88)	0.78 (0.61–1.00)
**Endocrine Society Guidelines**	**Cox proportional hazards model—hazard ratio (95% CI) of all participants (*n*** **=** 3311)			
25(OH) vitamin-D categories	Unadjusted	Model 1	Model 2	Model 3
<20	Referent	Referent	Referent	Referent
20–29.99	0.85 (0.68–1.06)	0.78 (0.62–0.98)	0.79 (0.63–1.00)	0.85 (0.68–1.07)
30+	0.52 (0.26–1.04)	0.47 (0.23–0.94)	0.49 (0.24–0.99)	0.57 (0.28–1.14)
**Cox proportional hazards model—hazard ratio (95% CI) of all participants (*n*** = 3311)				
Log-25(OH) vitamin-D per 1 unit SD	Unadjusted	Model 1	Model 2	Model 3
Continuous	0.94 (0.87–1.02)	0.89 (0.82–0.97)	0.90 (0.83–0.98)	0.96 (0.88–1.05)

Models:

Unadjusted (*n* = 3311).

Model 1: age, sex, education, occupation status (*n* = 3309).

Model 2: Model 1 + smoking, physical activity, alcohol use, and aldosterone (*n* = 3261).

Model 3: Model 2 + body-mass index (kg/m^2^) (*n* = 3261).

Interpretation: A 1-SD higher log-25(OH)D was associated with a 4% lower risk of incident diabetes (continuous association, Model 3, not significant).

### Association of total 25(OH)D with incident diabetes among participants with detectable 25(OH)D2 (*n* = 1671)

The unadjusted and adjusted HRs for incident diabetes associated with 25(OH)D in categories and log-25(OH)D SD among participants with detectable 25(OH)D2 at baseline are presented in Table 3. In fully adjusted models including BMI, 25(OH)D ≥20 ng/ml compared to <12 ng/ml was associated with a 36% (HR 0.64, 95% CI: 0.46, 0.91) lower risk of diabetes, whereas 25(OH)D ≥30 ng/ml compared to <20 ng/ml was associated with a 37% (HR 0.63, 95% CI: 0.29,1.34) nonsignificant lower risk of diabetes. Every 1-log SD increase in 25(OH)D was associated with a 13% (HR 0.87, 95% CI: 0.77–0.98) lower risk of diabetes with full adjustment, including BMI.

**Table 3:** The association of total 25(OH) vitamin D with incident diabetes among participants with detectable vitamin D2 and D3.

Institute of Medicine Guidelines	Cox proportional hazards model—hazard ratio (95% CI) of all participants (*n* = 1671)			
25(OH) vitamin-D categories	Unadjusted	Model 1	Model 2	Model 3
<12	Referent	Referent	Referent	Referent
12–19.9	0.88 (0.68–1.15)	0.82 (0.63–1.07)	0.85 (0.65–1.12)	0.89 (0.68–1.17)
20+	0.66 (0.48–0.91)	0.57 (0.41–0.80)	0.59 (0.42–0.84)	0.64 (0.46–0.91)
**Endocrine Society Guidelines**	**Cox proportional hazards model—hazard ratio (95% CI) of all participants (*n*** = 1671)			
25(OH) vitamin-D categories	Unadjusted	Model 1	Model 2	Model 3
<20	Referent	Referent	Referent	Referent
20–29.9	0.73 (0.54–0.99)	0.66 (0.49–0.90)	0.68 (0.49–0.92)	0.71 (0.52–0.97)
30+	0.58 (0.28–1.24)	0.53 (0.25–1.12)	0.56 (0.26–1.19)	0.63 (0.29–1.34)
**Cox proportional hazards model—hazard ratio (95% CI) of all participants (*n*** = 1671)				
Log-25(OH) vitamin D per 1 unit SD	Unadjusted	Model 1	Model 2	Model 3
Continuous	0.87 (0.78–0.97)	0.82 (0.73–0.92)	0.84 (0.74–0.95)	0.87 (0.77–0.98)

Models:

Unadjusted (*n* = 1671).

Model 1: age, sex, education, occupation status (*n* = 1670).

Model 2: Model 1 + smoking, physical activity, alcohol use, and aldosterone (*n* = 1648).

Model 3: Model 2 + body-mass index (kg/m^2^) (*n* = 1648).

Interpretation: A 1-SD higher log-25(OH)D was associated with a 13% lower risk of incident diabetes (continuous association, Model 3).

### Association of either 25(OH)D2 or 25(OH)D3 individually with incident diabetes (*n* = 1671, *n* = 3311)

A 1-SD increase in log-25(OH)D2 was associated with a 12% lower risk of incident diabetes prior to adjustment for BMI (Model 2) and 11% lower risk (HR 0.89, 95% CI: 0.79–1.01) after adjustment for BMI among 1671 participants with detectable 25(OH)D2 (Table 4). A 1-SD increase in log-25(OH)D3 was associated with a nonsignificant 7% lower risk (HR 0.93, 95% CI: 0.86, 1.01) of incident diabetes, which was further attenuated by adjustment for BMI (HR 0.99, 95% CI: 0.91, 1.08).

**Table 4:** The association of log-25(OH) D2 and D3 with incident diabetes.

Cox proportional hazards model—hazard ratio (95% CI) of all participants (*n* = 1671)				
Log-25(OH) vitamin D2 standard deviations	Unadjusted	Model 1	Model 2	Model 3
Continuous	0.91 (0.81–1.02)	0.88 (0.78–0.99)	0.88 (0.78–1.00)	0.89 (0.79–1.01)
**Cox proportional hazards model—hazard ratio (95% CI) of all participants (*n*** = 3311)				
Log-25(OH) vitamin D3 standard deviations	Unadjusted	Model 1	Model 2	Model 3
Continuous	0.96 (0.89–1.04)	0.92 (0.85–1.00)	0.93 (0.86–1.01)	0.99 (0.91–1.08)

Models:

Unadjusted (*n* = 1671, *n* = 3311).

Model 1: age, sex, education, occupation status (*n* = 1670, *n* = 3309).

Model 2: Model 1 + smoking, physical activity, alcohol use and aldosterone (*n* = 1648, *n* = 3261).

Model 3: Model 2 + body-mass index (kg/m^2^) (*n* = 1648, *n* = 3261).

Interpretation: A 1-SD higher log-25(OH)D3 was associated with a 1% lower risk of incident diabetes (Continuous association, Model 3, not significant).

### Interaction of vitamin-D-binding protein genotypes and total plasma 25(OH)D with incident diabetes (*n* = 3804)

The unadjusted and adjusted HRs for the continuous association of total plasma 25(OH)D with incident diabetes are presented alongside the *P* value for interaction by DBP genotype in Table 5. Both prior to and following adjustment, there was no evidence of effect modification by the rs7041 or rs4588 vitamin-D binding protein genotype.

**Table 5:** Hazard ratios of incident diabetes per 1 ng/ml higher total plasma 25-hydroxyvitamin D according to vitamin-D binding protein genotype (rs7041 and rs4588).

Interaction models	rs4588 (*n* = 1910)		
	AA/AC HR (95% CI)	CC HR (95% CI)	*P* for interaction
No. of events/*N*	57/388	274/1522	
Unadjusted	0.99 (0.95, 1.03)	0.98 (0.96, 1.00)	0.649
Model 1	0.98 (0.94, 1.02)	0.97 (0.95, 0.99)	0.617
Model 2	0.98 (0.94, 1.02)	0.97 (0.95, 0.99)	0.767
Model 3	0.98 (0.94, 1.02)	0.98 (0.96, 1.00)	0.975
**Interaction models**	**rs7041 (*n*** = 1894)		
	**GG/GT HR (95% CI)**	**TT HR (95% CI)**	***P*** **for interaction**
No. of events/*N*	114/556	217/1338	
Unadjusted	0.98 (0.95, 1.01)	0.98 (0.96, 1.00)	0.925
Model 1	0.97 (0.94, 1.00)	0.97 (0.95, 0.99)	0.997
Model 2	0.97 (0.94, 1.00)	0.97 (0.95, 0.99)	0.980
Model 3	0.98 (0.95, 1.01)	0.98 (0.96, 1.01)	0.664

Models:

Unadjusted (*n* = 1910, *n* = 1894).

Model 1: age, sex, education, occupation status (*n* = 1910, *n* = 1894).

Model 2: Model 1 + smoking, physical activity, alcohol use and aldosterone (*n* = 1910, *n* = 1894).

Model 3: Model 2 + body-mass index (kg/m^2^) (*n* = 1910, *n* = 1894).

## Discussion

In this prospective study of AA adults, we found that, among individuals without diabetes at baseline, higher log-25(OH)D, 25(OH)D ≥20 compared to <12 ng/ml and 25(OH)D ≥30 compared to <20 ng/ml were associated with a lower diabetes risk prior to adjustment for BMI. These findings were attenuated by adjustment for BMI, except when using IOM categorization. Among participants with detectable 25(OH)D2, higher log-25(OH)D and 25(OH)D ≥20 compared to <12 ng/ml were associated with a lower risk of diabetes, irrespective of BMI. These results suggest that higher levels of 25(OH)D may be protective against the development of diabetes among AA individuals with detectable 25(OH)D2, regardless of obesity status.

Consistent with observational studies in majority NHW cohorts [10–12], we found significant associations between increasing 25(OH)D and a lower risk of diabetes. Our results are consistent with an ancillary study of the Diabetes Prevention Program (57% NHW, 20% AA, and 23% Hispanic, Asian and American Indian) [33], where the highest tertile compared to the lowest tertile of 25(OH)D was associated with a 28% lower risk of developing diabetes with similar findings among the NHW and a non-NHW subgroup (AA, Hispanic, Asian and American Indian combined) [33].

Our results are divergent from two prior multi-ethnic analyzes. First, in the ARIC study, lower 25(OH)D was associated with a higher risk of incident diabetes in quintiles among NHWs, but not among AA participants [13]. Many of the AA participants in the ARIC study were also from the Jackson, MS tri-county area, and both cohorts had few participants with 25(OH)D in the “sufficient” range. Differences between the cohorts were that: (1) ARIC analyzed fewer AA participants (*n* = 2102) compared to 3311 in JHS; (2) in ARIC, there were only 97 AA participants in the 25(OH)D referent group (quartile 5), compared to 2,044 NHWs; and (3) ARIC used a more limited definition of diabetes than was used in the JHS, possibly excluding some incident cases.

Second, in the Third National Health and Nutrition Examination Survey, odds ratios for diabetes were lower in higher quartiles of 25(OH)D among NHWs and Hispanic Americans but not AA participants (*n* = 1736 AAs) [8]. This approach differed methodologically from our analysis. We categorized individuals prospectively by incident diabetes status using time-to-event data. We also used a more comprehensive definition of diabetes that included HbA1c. Also, there are potential differences in 25(OH)D by geographic region of the United States, based on the level of UV exposure and AA genetic admixture [1, 34].

This study suggests higher levels of 25(OH)D may lower the risk of diabetes in AA populations. Serum 25(OH)D levels are increased readily through vitamin D3 supplementation and may present an effective, broad-scale, low-cost opportunity for diabetes prevention in the AA community. However, results of two recent randomized controlled trials of vitamin-D supplementation show no statistically significant difference in progression to diabetes between supplement and placebo groups of individuals with prediabetes at study inception recruited irrespective of vitamin-D deficiency [35, 36]. The D2d study conducted by Pittas et al. found that individuals receiving 4000 IU/day had a lower, but not statistically significant, risk of incident diabetes over a median follow-up time of 2.5 years. Approximately 25% of the participants were black. Although not statistically significant, subgroup analysis showed black participants had a lower hazard ratio compared to white and other race participants. Notably, low 25(OH)D was not an inclusion criterium for the study nor was it powered to detect risk reductions less than 25% [35]. Measures of glucose control and insulin resistance were improved in participants with prediabetes in the Tromsø Study, receiving 20,000 IU of vitamin D weekly for 5 years compared to placebo. In addition, the conversion rate to diabetes was reduced in the treatment compared to the placebo group. These differences were not statistically significant, and the study was not powered to detect conversion rate reductions less than 30% [36]. No randomized, controlled trial of vitamin-D supplementation has been conducted in an AA population with 25(OH)D deficiency for ethical reasons.

Testing for effect modification of the relationship between 25(OH)D and incident diabetes by DBP genotype did not yield significant differences. Previous investigations have found associations between single-nucleotide polymorphisms (rs7041 and rs4588) and increased risk and odds of incident and prevalent diabetes, respectively [37, 38], and efficacy of response to vitamin-D supplementation [39]. This suggests that the role of DBP genotype in glucose metabolism is at least partially distinct from its sterol-binding-related activity and should be further investigated prospectively in AA populations.

### Detectable 25(OH)D2 and 25(OH)D3 and glucose metabolism

The participants in our study with detectable 25(OH)D2 had a 2.7 ng/ml higher 25(OH)D with slightly better dietary intake (Supplemental Table 2) [18]. Estimates from the National Health and Nutrition Examination Survey reveal that 25(OH)D2 is detectable in only 19% of the US population and skews towards older individuals potentially taking vitamin-D supplements [40]. Ninety-nine percent of 25(OH)D is bound to DBP. Although 25(OH)D2 represents a smaller fraction of overall 25(OH)D compared to 25(OH)D3, it binds DBP with less avidity and may be more bioavailable [2]. In our study, 25(OH)D2 was detectable in 50% of participants. Among those participants, stronger associations of 25(OH)D with incident diabetes existed. Thus, it is paramount to investigate the role of 25(OH)D2, 25(OH)D3, and total 25(OH)D in glucose metabolism.

### Obesity and vitamin D

Currently, obesity impacts over 1 in 3 Americans, with a higher prevalence in the AA population [41]. In our cohort, the average BMI was 31.1 kg/m^2^ at baseline, consistent with US representative samples [41]. Higher BMI is associated with lower 25(OH)D, with disputed biological mechanisms [42, 43]. The classical theory is sequestration of 25(OH)D3 into body fat compartments as described by Wortsman et al. [42]. Drincic et al. postulated volumetric dilution, rather than sequestration, is the cause of low 25(OH)D in obese individuals [43]. Other studies suggest other mechanisms, including decreased expression of enzymes involved in vitamin-D metabolism, including hepatic 25-hydroxylase, cutaneous and visceral adipose tissue 25- and 1-alpha hydroxylase, in obese individuals [44, 45]. Vimaleswaran et al. used bi-directional genetics to study the direction of causality for BMI and 25(OH)D, finding directionality favored higher BMI leading to lower 25(OH)D [46]. A study of 25(OH)D and adiposity among AA and Hispanic American participants revealed an inverse cross-sectional association of 25(OH)D with BMI, but no longitudinal association with change in adiposity measures [47]. In this study, BMI attenuated the findings in the main analysis, but among those with detectable 25(OH)D2, results remained significant with only mild attenuation.

### Vitamin-D guidelines

Controversy exists regarding healthy 25(OH)D concentration. The IOM and ES guidelines used similar mineral metabolism outcomes, yet reached different conclusions for levels of 25(OH)D deficiency, insufficiency, and sufficiency [22]. The target population drives these differences, as the IOM guideline focused on vitamin D for the general population, and the ES guideline targeted diseased and high-risk clinic populations [48, 49]. Both guidelines were used in this analysis. We found that the IOM categorization may be more appropriate when considering diabetes risk in AA populations, as the prevalence of 25(OH)D ≥30 is negligible (<3%) in this cohort. Presuming that 25(OH)D is reflective of bioavailable vitamin D in AA populations, which has been recently challenged [16], approximately 80% of participants in JHS had 25(OH)D in the deficient range, <20 ng/ml, according to ES guidelines [23]. Vitamin-D production is reduced by melanin in the skin; therefore, AA individuals have lower synthesis of vitamin D for equivalent sun exposure [50]. If there is a threshold level for glycemic benefits, as suggested by Sorkin et al. [51], it may be difficult to detect a benefit among AA individuals, especially when using the higher ES cutoff for sufficiency.

### Strengths and limitations

The strengths of our study include a large, socioeconomically diverse, AA cohort. JHS used liquid-based tandem mass spectroscopy—the gold standard—to measure 25(OH)D2, 25(OH)D3 and total 25(OH)D at baseline. JHS used validated questionnaires and a comprehensive documentation of diabetes over time, including fasting glucose, HbA1c, and medication use with improved detection of incident diabetes cases using American Diabetes Association criteria. Despite these strengths, there are some limitations. First, participants in JHS are from one geographic area in the southeastern U.S. and may not be representative of all AA individuals. Second, JHS did not measure vitamin-D binding protein levels, thus was unable to assess bioavailable vitamin D. Third, JHS measured serum 25(OH)D concentrations at only a single time and thus was unable to determine whether changes in 25(OH)D occurred during the follow-up period. Fourth, participants with detectable 25(OH)D2 had a slightly better ideal dietary intake based on American Heart Association’s Life’s Simple 7 classification with 0.4% and 1% in the ideal category for those without vs. with detectable 25(OH)D2 (Supplemental Table 2). Thus, differences in diet may have made a minor contribution to differences in incident diabetes. Finally, only 13 individuals reported taking vitamin-D supplements. This may be due to underreporting, and others in the JHS may have taken multivitamins containing vitamin D.

## Conclusion

This study suggests that higher levels of 25(OH)D may be associated with a lower risk of diabetes in AA populations, particularly those with detectable 25(OH)D2. Given the increased prevalence of diabetes in the AA population, it is important to develop biomarkers of diabetes risk and targets for diabetes prevention therapies. Thus the further investigation of the role of vitamin D in diabetes is warranted to advance diabetes equity.

## Supplementary information

Supplement

Supplemental Methods

## Data Availability

Restrictions apply to the availability of data generated or analyzed during this study to preserve patient confidentiality or because they were used under license. The corresponding author will on request detail the restrictions and any conditions under which access to some data may be provided.

## Code availability

The data, analytical methods, code, and study materials are available to other researchers for purposes of reproducing the results or replicating the procedure by following the JHS publication procedures and data-use agreement guidelines [52].
