# Supplementary material for: The association of serum vitamin D with incident diabetes in an African American population
Source: Nutr Diabetes. 2022 Oct 13;12:43. doi: 10.1038/s41387-022-00220-4 (PMC9562299; doi:10.1038/s41387-022-00220-4)

The Association of Serum Vitamin D with Incident Diabetes in an African American Population

Joshua J. Joseph, MD (1), Susan Langan, MS (2), Joseph Lunyera, MD (4), Bjorn Kluwe, BS (1), Haiying Chen, MD, Ph.D. (3), Michael C. Sachs, Ph.D. (5), Kristin G. Hairston, MD, MPH (3), Alain G. Bertoni, MD (3), Willa A. Hsueh, MD (1) and Sherita H. Golden, MD, MHS (2)

1. Division of Endocrinology, Diabetes and Metabolism, The Ohio State University Wexner Medical Center, Columbus, Ohio, USA
2. Division of Endocrinology, Diabetes and Metabolism, Johns Hopkins University School of Medicine, Baltimore, Maryland, USA
3. Division of Public Health Sciences, Wake Forest School of Medicine, Winston-Salem, North Carolina, USA (HC, KGH, AGB)
4. Unit of Biostatistics, Duke University School of Medicine, Durham, North Carolina, USA (JL)
5. Department of Medical Epidemiology and Biostatistics, Karolinska Institutet, Stockholm, Sweden

Supplemental Tables and Figures

Supplemental Table 1. Baseline Characteristics of Jackson Heart Study Participants by 25(OH)D Categories (Endocrine Society)

(Online Supplemental Material)

|  | | Vitamin D Concentration (ng⁄mL) | | |  |
| --- | --- | --- | --- | --- | --- |
| Baseline Characteristics^1^ | All n = 3311 | Less than 20 n = 2648 | 20-29.9 n = 584 | 30+ n = 79 | p-value |
| Age | 53.31 (12.48) | 52.18 (12.33) | 57.61 (12.07) | 59.20 (12.2) | <0.0001 |
| Female, sex | 63.30 | 65.52 | 52.57 | 68.35 | <0.0001 |
| Education > Bachelor's Degree | 37.03 | 36.06 | 40.92 | 40.51 | 0.0719 |
| Occupation, Management/Professional | 38.95 | 38.33 | 42.10 | 36.71 | 0.2213 |
| Current Smoking | 11.96 | 12.82 | 8.97 | 5.13 | 0.0059 |
| Current Alcohol Use | 49.98 | 50.15 | 48.97 | 51.90 | 0.8244 |
| AHA Physical Activity^2^ |  |  |  |  | <0.0001 |
| Poor | 45.05 | 46.75 | 38.94 | 32.91 |  |
| Intermediate | 33.53 | 33.69 | 34.13 | 24.05 |  |
| Ideal | 21.42 | 19.56 | 26.93 | 43.04 |  |
| AHA Dietary Intake^2^ |  |  |  |  | <0.0001 |
| Poor | 63.79 | 66.28 | 54.62 | 48.10 |  |
| Intermediate | 35.52 | 33.23 | 43.84 | 50.63 |  |
| Ideal | 0.69 | 0.49 | 1.54 | 1.27 |  |
| Vegetables (servings/day) | 1.46 (0.69) | 1.46 (0.7) | 1.46 (0.71) | 1.38 (0.45) | 0.9677 |
| Fruit (servings/day) | 1.53 (1.17) | 1.50 (1.18) | 1.63 (1.15) | 1.48 (0.85) | 0.0037 |
| Body-mass Index (kg/m^2^)^3^ | 31.20 (7.04) | 31.66 (7.27) | 29.45 (5.72) | 28.50 (5.03) | <0.0001 |
| Waist circumference (cm)^4^ | 98.65 (15.64) | 99.34 (16.08) | 96.43 (13.38) | 91.66 (12.48) | <0.0001 |
| Systolic blood pressure (mmHg) | 124.97 (17.43) | 124.59 (17.33) | 126.46 (17.55) | 126.58 (19.46) | 0.0681 |
| Diastolic blood pressure (mmHg) | 79.37 (10.37) | 79.49 (10.43) | 78.88 (10) | 78.91 (11.18) | 0.2597 |
| Glucose (mg/dl) | 90.30 (8.9) | 90.30 (8.96) | 90.45 (8.78) | 89.18 (8.02) | 0.7902 |
| Hemoglobin A1c %^5^ | 5.50 (0.47) | 5.49 (0.47) | 5.53 (0.43) | 5.47 (0.46) | 0.6230 |
| ln HOMA-IR^6^ | 1.11 (0.55) | 1.14 (0.55) | 1.03 (0.56) | 0.94 (0.5) | <0.0001 |
| ln HOMA-β^6^ | 5.28 (0.51) | 5.30 (0.51) | 5.18 (0.5) | 5.16 (0.47) | <0.0001 |
| Aldosterone (ng/dl) | 5.49 (4.6) | 5.38 (4.7) | 5.88 (4.15) | 6.16 (3.91) | <0.0001 |
| hs-CRP (mg/dl)^7^ | 0.47 (0.71) | 0.48 (0.71) | 0.42 (0.72) | 0.51 (0.71) | 0.0349 |
| Diabetes Incidence Rate /1000 PY | 24.33 | 25.26 | 21.71 | 13.33 |  |

Legend Supplemental Table 1:

| ^1^ Mean (SD) or percentages are listed, P-values were calculated using 2 (categorical variables), ANOVA (parametric continuous variables), and Kruskal-Wallis test (nonparametric continuous variables). |
| --- |
| ^2^ Physical activity and dietary intake recommendations were defined by AHA 2020 guidelines. Physical activity was considered ideal if participant achieved 150 min/wk or greater of moderate-intensity or 75 min/wk or greater of vigorous-intensity physical activity.^22^ Dietary intake was considered ideal if participant met four to five of the five following recommendations: fruits and vegetables of 4.5 cups/d or more; fish of two 3.5-oz servings per week or more (preferably oily fish); fiber-rich whole grains of three 1-oz-equivalent servings per day or more; sodium 1500 mg/d or less; and sugar-sweetened beverages of 450 kcal (36 oz)/wk or less.^22^ |
| ^3^ body-mass index – n= 3311 (<12 n=2648, 12-19.9 n=584, 20+ n=79) |
| ^4^ waist circumference – n= 3311 (<12 n=2648, 12-19.9 n=584, 20+ n=79) |
| ^5^ hemoglobin A1c – n= 3261 (<12 n=2609, 12-19.9 n=575, 20+ n=77) |
| ^6^ HOMA-IR and HOMA-β – n= 3188 (<12 n=2551, 12-19.9 n=561, 20+ n=76) |
| ^7^ high sensitivity-C reactive protein – n= 3310 (<12 n=2647, 12-19.9 n=584, 20+ n=79) |

Supplemental Table 2. Baseline Characteristics of Jackson Heart Study Participants by 25(OH) Vitamin D2 Categories

(Online Supplemental Material)

| Baseline Characteristics^1^ | All n = 3311 | VitD2 Undetectable n = 1640 | VitD2 Detectable n = 1671 | p-value |
| --- | --- | --- | --- | --- |
| Age | 53.31 (12.48) | 52.42 (12.2) | 54.18 (12.7) | <0.0001 |
| Female, sex | 63.30 | 61.10 | 65.47 | 0.0091 |
| Education > Bachelor's Degree | 37.03 | 35.30 | 38.72 | 0.0419 |
| Occupation, Management/Professional | 38.95 | 36.61 | 41.26 | 0.0061 |
| Current Smoking | 11.96 | 13.76 | 10.20 | 0.0017 |
| Current Alcohol Use | 49.98 | 51.56 | 48.44 | 0.0729 |
| AHA Physical Activity^2^ |  |  |  | 0.3151 |
| Poor | 45.05 | 45.09 | 45.00 |  |
| Intermediate | 33.53 | 34.47 | 32.62 |  |
| Ideal | 21.42 | 20.44 | 22.38 |  |
| AHA Dietary Intake^2^ |  |  |  | 0.0068 |
| Poor | 63.79 | 65.85 | 61.76 |  |
| Intermediate | 35.52 | 33.78 | 37.22 |  |
| Ideal | 0.69 | 0.37 | 1.02 |  |
| Vegetables (servings/day) | 1.46 (0.69) | 1.46 (0.69) | 1.46 (0.7) | 0.5600 |
| Fruit (servings/day) | 1.53 (1.17) | 1.53 (1.25) | 1.52 (1.08) | 0.1715 |
| Body-mass Index (kg/m^2^)^3^ | 31.20 (7.04) | 31.45 (7.31) | 30.94 (6.75) | 0.0841 |
| Waist circumference (cm)^4^ | 98.65 (15.64) | 98.96 (16.04) | 98.34 (15.22) | 0.2358 |
| Systolic blood pressure (mmHg) | 124.97 (17.43) | 124.93 (17.71) | 125.00 (17.16) | 0.8398 |
| Diastolic blood pressure (mmHg) | 79.37 (10.37) | 79.56 (10.58) | 79.18 (10.16) | 0.3596 |
| Glucose (mg/dl) | 90.30 (8.9) | 90.67 (8.96) | 89.94 (8.84) | 0.0301 |
| Hemoglobin A1c %^5^ | 5.50 (0.47) | 5.49 (0.47) | 5.51 (0.46) | 0.3386 |
| ln HOMA-IR^6^ | 1.11 (0.55) | 1.12 (0.54) | 1.10 (0.57) | 0.6067 |
| ln HOMA-β^6^ | 5.28 (0.51) | 5.27 (0.51) | 5.29 (0.52) | 0.1955 |
| Aldosterone (ng/dl) | 5.49 (4.6) | 5.34 (4.12) | 5.63 (5.01) | 0.0422 |
| hs-CRP (mg/dl)^7^ | 0.47 (0.71) | 0.45 (0.63) | 0.48 (0.78) | 0.7210 |
| 25(OH) Vitamin D2 (ng/ml) | 2.53 (3.87) | -- | 2.53 (3.87) |  |
| 25(OH) Vitamin D3 (ng/ml) | 13.56 (5.97) | 13.46 (6.06) | 13.67 (5.89) | 0.1951 |
| Total 25(OH) Vitamin D (ng/ml) | 14.84 (6.5) | 13.46 (6.06) | 16.20 (6.64) | <0.0001 |
| Diabetes Incidence Rate /1000 PY | 24.33 | 24.23 | 24.42 |  |

Legend Supplemental Table 2:

| ^1^ Mean (SD) or percentages are listed, P-values were calculated using 2 (categorical variables), ANOVA (parametric continuous variables), and Kruskal-Wallis test (nonparametric continuous variables). |
| --- |
| ^2^ Physical activity and dietary intake recommendations were defined by AHA 2020 guidelines. Physical activity was considered ideal if participant achieved 150 min/wk or greater of moderate-intensity or 75 min/wk or greater of vigorous-intensity physical activity.^22^ Dietary intake was considered ideal if participant met four to five of the five following recommendations: fruits and vegetables of 4.5 cups/d or more; fish of two 3.5-oz servings per week or more (preferably oily fish); fiber-rich whole grains of three 1-oz-equivalent servings per day or more; sodium 1500 mg/d or less; and sugar-sweetened beverages of 450 kcal (36 oz)/wk or less.^22^ |
| ^3^ body-mass index – n= 3311 (D2 Undetectable n=1640, D2 Detectable n=1671) |
| ^4^ waist circumference – n= 3311 (D2 Undetectable n=1640, D2 Detectable n=1671) |
| ^5^ hemoglobin A1c – n= 3261 (D2 Undetectable n=1613, D2 Detectable n=1648) |
| ^6^ HOMA-IR and HOMA-β – n= 3188 (D2 Undetectable n=1583, D2 Detectable n=1605) |
| ^7^ high sensitivity-C reactive protein – n= 3310 (D2 Undetectable n=1639, D2 Detectable n=1671) |


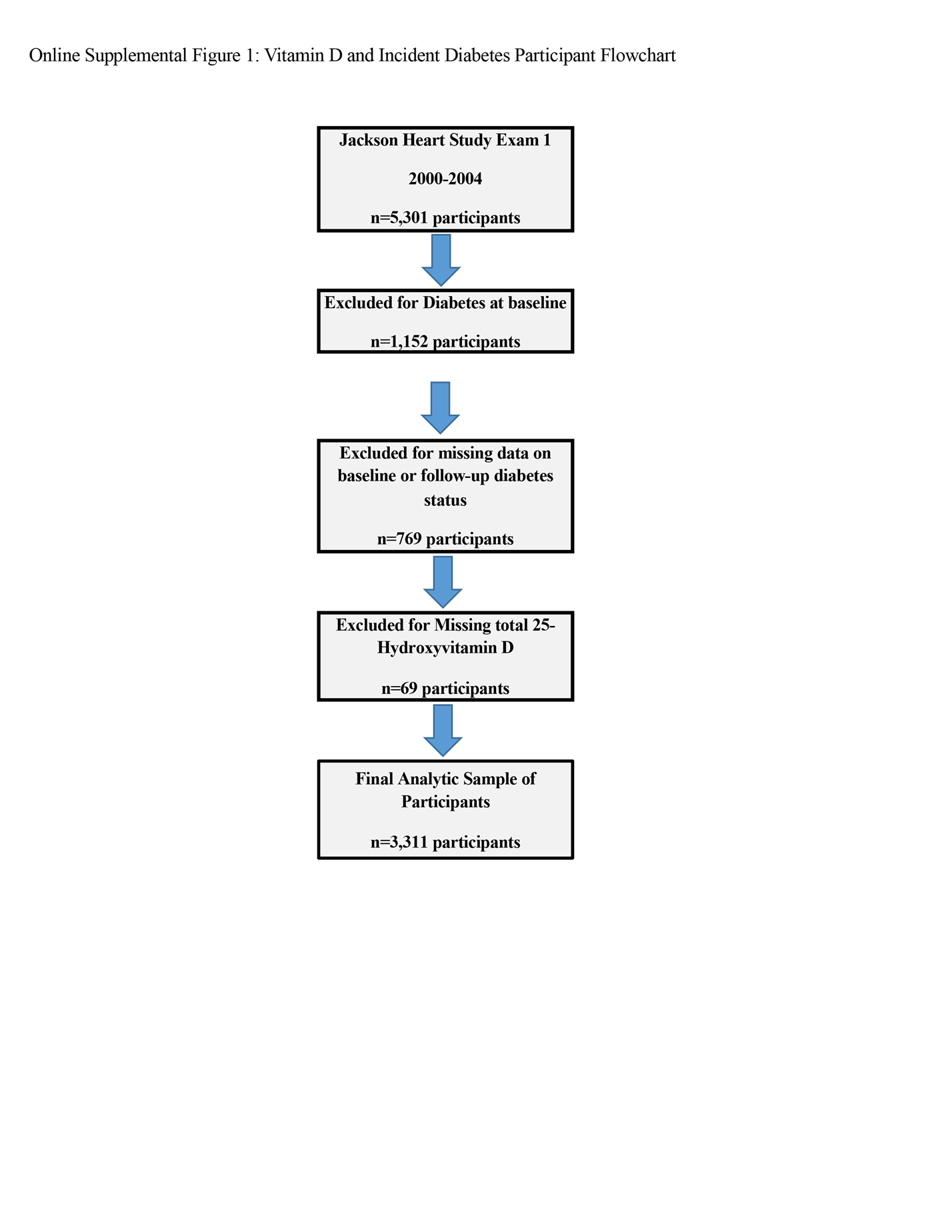

Supplement: Supplementary file 1 — Supplement [file 41387_2022_220_MOESM1_ESM.docx]
