# Supplementary material for: The association of serum vitamin D with incident diabetes in an African American population
Source: Nutr Diabetes. 2022 Oct 13;12:43. doi: 10.1038/s41387-022-00220-4 (PMC9562299; doi:10.1038/s41387-022-00220-4)
Supplement: Supplementary file 2 — Supplemental Methods [file 41387_2022_220_MOESM2_ESM.docx]

Supplemental Methods:

***Quantification of 25-hydroxy vitamin D2 [25(OH)D2], 25-hydroxy vitamin D3 [25(OH)D3], and epimeric vitamin D3 [epi-25(OH)D3] in human serum or plasma using liquid chromatography-tandem mass spectrometry (LC-MS/MS)(20)***

Patient serum, calibrators, and controls (200 μL) were alkalinized with 200 μL 1 N sodium hydroxide in a 96-deep well plate, covered with a silicone cover (MicroMat, Varian) vortexed for 15 s, and incubated at room temperature for 15 min. Internal standard [200 μL; 121.3 nmol/L (50 ng/mL) 25(OH)D2 and 25(OH)D3 in methanol] was added to each well and the plates were covered and vortexed for 15 sec. Samples were extracted with 1 ml n-heptane (5 min vortex, covered) and the plates were centrifuged at 1,100xg for 4 min at room temperature in a Beckman Allegra X-22 centrifuge equipped with a 96-well plate rotor. To seal the two plates together, a transfer gasket and another 96-deep well plate were fitted on top of the extraction plate. The sealed plates, held together by the gasket, were then placed in a dry ice-acetone bath for 50 min to freeze the lower aqueous layer. The entire organic layer was then transferred to the new plate by inverting and gently tapping the assembly on the benchtop. The extracts were dried under forced nitrogen at room temperature (Turbovap) and the residue was reconstituted in 200 μL 75% methanol in water.

In addition, after centrifuging the 96-well plate, the upper layer were removed using the automated liquid handler rather than using the liquid transfer gasket. A portion of the dissolved extracts (40 μL) was injected and developed using penta-fluorophenyl propyl chromatography (Restek PFPP, 100x3.2 cm 5 µm, 3.5 μm column with an integrated guard column) with isocratic mobile phase (2 mM ammonium acetate, 0.1% formic acid in 78.8% methanol in water, 0.4 mL/min) and analyzed using isotope dilution-tandem mass spectrometry (Quattro Micro with a 2795 HPLC, Waters). Concentrations of 25-hydroxy D2 and D3 were then calculated using a calibration curve made in 2% bovine serum albumin. Two blinded control samples were interspersed in each batch.
